# A comparison of lower body gait kinematics and kinetics between Theia3D markerless and marker-based models in healthy subjects and clinical patients

**DOI:** 10.1038/s41598-024-80499-8

**Published:** 2024-11-25

**Authors:** Sonia D’Souza, Tobias Siebert, Vincent Fohanno

**Affiliations:** 1 Gait laboratory, Olgahospital/Frauenklinik, Orthopaedic Clinic, Klinikum Stuttgart, Kriegsbergstrasse 62, 70174 Stuttgart, Germany; 2 Motion and Exercise Science, University of Stuttgart, Stuttgart, Germany; 3 Stuttgart Center of Simulation Science, University of Stuttgart, Stuttgart, Germany; 4 Qualisys AB, Kvarnbergsgatan 2, Gothenburg, 411 06 Sweden

**Keywords:** 3D motion capture, Pathological gait, Kinematics, Hip rotation, Knee rotation, Hereditary Motor and sensory neuropathy, Bone quality and biomechanics, Musculoskeletal system

## Abstract

Three-dimensional (3D) marker-based motion capture is the current gold standard to assess and monitor pathological gait in a clinical setting. However, 3D markerless motion capture based on pose estimation is advancing into the field of gait analysis. This study aims at evaluating the lower-body 3D gait kinematics and kinetics from synchronously recorded Theia3D markerless and CAST marker-based systems. Twelve healthy individuals and 34 clinical patients aged 8–61 years walked at self-selected speed over a 13 m long walkway. Similarity between models was statistically analysed using inter-trial variability, root mean square error, Pearson’s correlation coefficient and Statistical Parametric Mapping. Inter-trial variability was on average higher for clinical patients in both models. Overall, the markerless system demonstrated similar gait patterns although hip and knee rotations were non-comparable. Pelvic anterior tilt was significantly underestimated. Significant differences especially in peak values at specific phases of the gait cycle were observed across all planes for all joints (more so for clinical patients than healthy subjects) as well as in the sagittal powers of the hip, knee and ankle. Theia3D markerless system offers great potential in gait analysis. This study brings awareness to potential clinical users and researchers where they can have confidence, as well as areas where caution should be exercised.

**Supplementary Information:**

The online version contains supplementary material available at 10.1038/s41598-024-80499-8.

## Introduction

The analysis of gait in a clinical setting encapsulates quantitative measurement, identification of pathologies, interpretation and postulation of primary and secondary causes, and recommendation of treatment^1^. Three-dimensional (3D) kinematics are one of the fundamental parameters included in quantitative gait data and are captured by means of the well-established tool, namely, marker-based motion capture. Clad only in necessary or tight-fitting clothing, three or more passive markers per segment are attached at specific non-collinear anatomical landmarks on the skin. Cameras placed at strategic locations to optimise coverage of the markers then capture the marker positions during a calibration trial as well as the marker trajectories as the patient walks within the capture volume.

The markers in the calibration trial are used to define local coordinate systems for each body segment. Using these, the position and orientation of the segments at each frame of movement are estimated and kinematics then extracted^2^. With the help of 6 degrees of freedom inverse dynamics, kinematic data along with ground reaction vectors and anthropometric parameters are used to estimate internal joint moments and powers^3^.

Marker-based motion capture is not without its drawbacks. Skin marker-placement requires expertise in human anatomy and palpation. Misplacement can bring about a miscalculation in joint centres and coordinate systems, thereby causing relevant changes in kinematics, kinetics and powers, hence misrepresenting an individual’s gait patterns^4–6^. This can lead to possible misinterpretation of pathological gait^7,8^. There is a risk of markers falling off especially with younger children, in subjects with sweaty/clammy skin or in case of subjects with gait deficits such as foot drag, knock-kneed gait or scissor-gait. Some marker-based models use clusters affixed by means of super-wrap for dynamic tracking of segments^9^. In overweight patients, friction between the thighs could cause clusters to loosen. The operator has to be constantly attentive and re-affix the marker or cluster in exactly the same position. Another important drawback related to marker-based analysis is the movement between skin markers and underlying bone which creates soft tissue artefacts^10,11^. These can be reduced but not removed by defining multibody lower limb kinematic models optimization processes that include setting joint kinematic constraints^12^. Finally, patients who are physically weak, very young or non-compliant often cannot avail of the benefits of a 3D-analysis because of the long marker-preparation time.

Markerless motion capture by means of monocular or multiple video cameras has come a long way during the past three decades owing to developments in computer vision and machine learning. Its applications that were traditionally surveillance, film and gaming are now shifting towards biomechanics, medical and sports applications^13^. Two dimensional monocular systems using software such as DeepLabCut and OpenPose and hardware ranging from camcorders, Kinect Xbox, Biostage™ are available^14–16^. These solutions, whilst being low cost and space efficient are fraught with problems such as self-occlusion, missing joint centre locations and limitations when it comes to measuring joint angles in 3D^17^.

Multi-camera markerless motion capture tackles these issues by taking advantages of multiple views to estimate the skeleton pose. The most recent solutions show the use of neural networks to detect anatomical landmarks on each camera view to perform 3D pose estimation^14,17–20^. Other solutions such as “The Captury” and Simi base their pose estimation on more traditional methods, e.g. silhouette tracking, alone or combined with neural networks.

Theia3D (Theia Markerless Inc., Kingston, ON, Canada), a multi-camera approach, which uses a proprietary pose estimation algorithm is the focus of this study. The pose estimation based on image detection of anatomical landmarks and not on the tracking of markers on the skin could reduce the problem of soft tissue artefacts that affects marker-based data capture, but is yet to be demonstrated^17^. While the markerless methodology reduces the practical constraints mentioned earlier with marker-based motion capture, it is of great importance for researchers and clinicians to evaluate this approach by comparing it with the standard marker-based approach. If proven for reliability and repeatability, it could provide researchers and clinicians with a technology that facilitates the study and interpretation of biomechanics of normal and pathological human movement.

Some studies have assessed the Theia3D markerless system in healthy individuals. Kanko et al. reported a slightly higher inter-trial variability and lower inter-session gait kinematic variability compared to other marker-based methods^21^. Gait kinematics from synchronously captured markerless and marker-based data were compared and similar global segment pose estimates of thigh, shank and foot found except for the transverse plane components of the thigh and shank^22^. Good to excellent agreement of spatiotemporal gait parameters compared with a marker-based system and a gait mat system were also demonstrated^23^ and reproduced using a pressure-sensitive walkway^24^. Tang et al. detected significant differences in peak magnitudes of joint moments and powers when comparing Theia3D with a marker-based system during treadmill running^25^. Ito et al. compared the kinematics of walking, squatting and forward hopping between Theia3D and a marker-based system, reporting strong agreement in the sagittal plane, more specifically for the knee, ankle, and hip joints^26^. However, the findings were based on only three subjects and were inconclusive regarding the frontal and transversal planes for the same joints. None of these studies have presented the kinematics of the pelvis segment.

Very little is known about how pathological movement patterns of subjects with orthopaedic and/or neuro-orthopaedic disorders are reconstructed using markerless motion capture including Theia3D^27^. To the best of our knowledge only one study so far has compared the kinematics, including that of the pelvis, between Theia3D markerless and the conventional marker-based gait model in 36 patients with a variety of disorders^28^. Similar patterns between both systems were found albeit with offsets between most signals that contributed to a higher root mean square difference of > 6°. A limitation of this study was that both systems were not synchronised meaning that the markerless and markerbased data were not extracted from the same trials or strides.

The aim of this study is to enable clinicians and researchers make informed decisions on the usage of the Theia3D markerless system by determining its agreement with instrumented marker-based system in patients with orthopaedic and neuro-orthopaedic disorders undergoing a clinical gait analysis. This study is the first to fully assess the markerless technology for gait analysis in the same environment as (1) it assesses both healthy and clinical patients, (2) focuses on kinematics and kinetics and (3) captures data synchronously in both systems.

## Methods

### Experimental setup

The gait laboratory at Olgahospital (Stuttgart, Germany) is fitted with a marker-based system comprising eleven Oqus 400 cameras and two Oqus 201c video overlay cameras (Qualisys AB, Gothenburg, Sweden), synchronised with three force plates (Bertec Corp., Ohio, USA). The 3D cameras are wall-mounted at a height of either 1.8–2.5 m. The two video-overlay cameras are mounted at 0.7 m height, to record the frontal and sagittal plane and used as a visualisation tool for quality assurance and setting of gait events of the marker-based system.

The markerless system was borrowed from Qualisys AB for a period of four weeks and included eight Miqus video cameras wall-mounted at a height of 1.8–2.5 m and synchronised with the existing marker-based system. The experimental setup allowed for a simultaneous, trigger-generated data recording of both systems. Ground reaction forces were captured at 500 Hz sampling frequency.

### Participants

Participants included all clinical patients who visited the lab during the four-week loan period of the markerless system. Additionally, 12 healthy subjects (4:8 males: females aged 8–61 years) free of neuro-orthopaedic diseases were recruited for the study. Patients with abnormal gait patterns are referred internally to the lab for an analysis with concrete questions just as pre-operative diagnostics, therapy planning or orthotic testing. A sample size of 34 clinical patients (24:10 males: females aged 7–55 years) with a range of orthopaedic or neuro-orthopaedic disorders were evaluated. The breakup of the patient diagnoses was 64% neuro-orthopaedic disorders (30% cerebral palsy, 34% others such as hereditary sensory motor neuropathy, microcephalus, hydrocephalus etc.), and 36% orthopaedic ailments (of which 30% were flat-feet). 29 of the 34 patients were bilaterally affected, the remaining 5 patients exhibited compensatory gait deviations on the unaffected side. For a detailed break-up of the diagnosis, please see Supplementary Table S1.

**Fig. 1:**
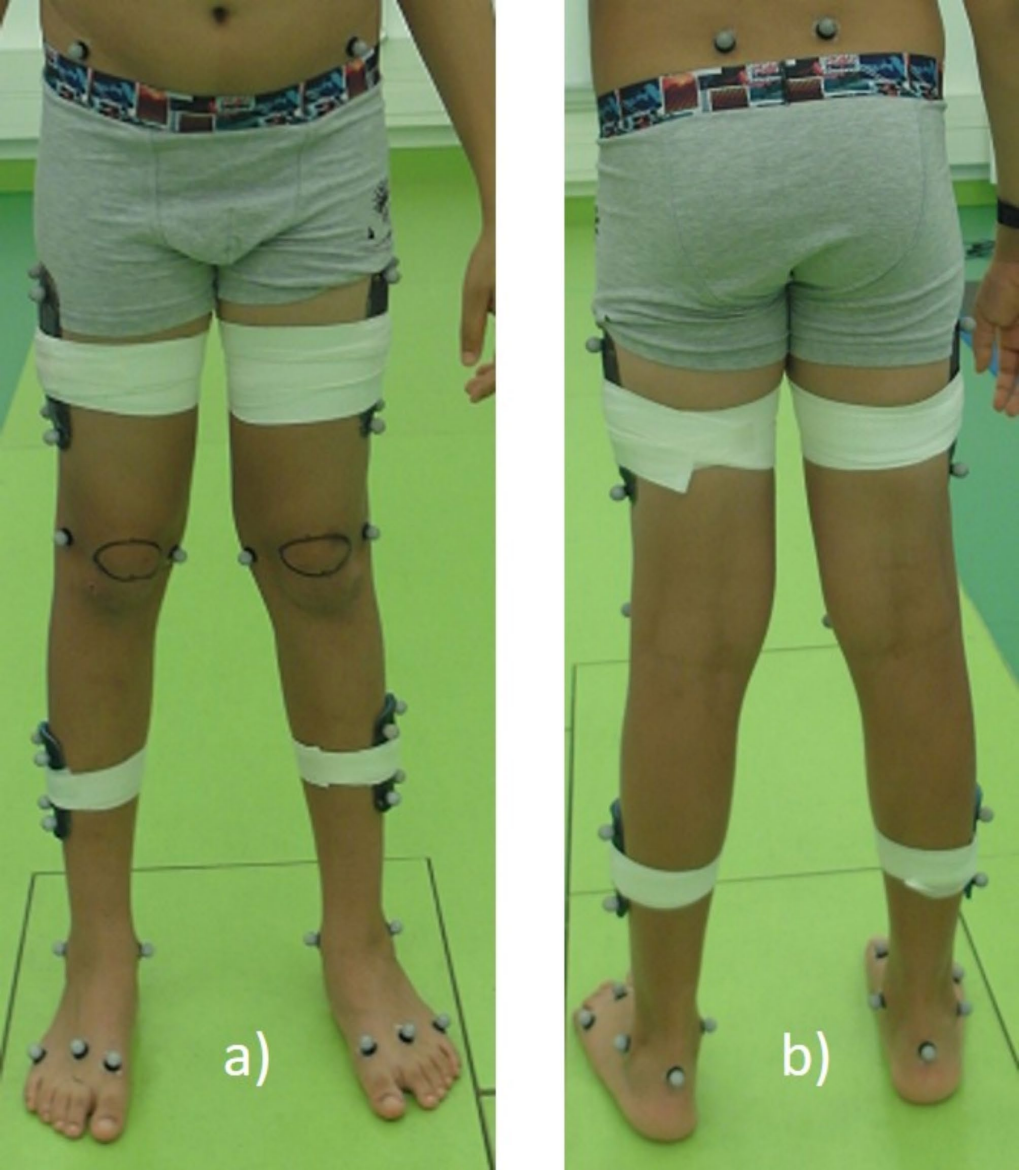
View from front (**a**) and back (**b**) on marker locations of the CAST lower body model^9^.

The experimental protocol for both healthy subjects and patients was approved by Klinikum Stuttgart (Olgahospital), Germany. Experiments were performed in accordance with standard operating procedures as part of routine clinical gait analysis in the orthopaedic department of Klinikum Stuttgart. The data was collected in compliance with ethical principles of the Declaration of Helsinki. Detailed information about the measurement procedure was given and signed consent was obtained from the participant or, in case of a minor, from the accompanying parent with regards to collection, analysis and publication for this non-interventional study. Data was captured by the same team of physiotherapists and lab engineer, all experienced with clinical gait analysis. Participants were barefoot and wore minimal, tight-fitting clothing so that markers and clusters could be attached on the skin. Specific anatomical positions were palpated and retro-reflective markers affixed as per the CAST model on lower-body landmarks bilaterally (Fig. 1)^9^.

One static calibration trial, required solely for the marker-based system, was recorded with the subject standing in the middle of the capture volume. As per standard operating procedure during a clinical gait analysis, six walking trials at self-selected speed over the 13 m long walkway were synchronously tracked in both systems via the Qualisys Track Manager Software (v2021.1 build 6470). At the start of each trial, the starting point was adjusted medio-laterally and/or anterior-posteriorly in order to obtain as many clean foot strikes over the force plates as possible.

### Data analysis

A total of 6 full gait cycles per leg for each participant was analysed. Marker trajectories were filtered at 6 Hz, ground reaction forces at 25 Hz by means of a 2nd order low-pass Butterworth filter in Visual3D (v2020.11.2). The Visual3D segment optimization procedure (six degrees of freedom for each segment) was used for the inverse kinematics to compute the joint angles where all markers were assigned the same weight. Kinematics of two consecutive gait cycles per trial were analysed. The number of cycles for the kinetics was lower since not all subjects, especially children, could hit the force plates cleanly in all trials, even after trying to adjust the position at which they started walking.

Markerless data were exported from Qualisys Track Manager to Theia3D (v2022.1.0.2309) for processing where they were filtered at 6 Hz by means of Generalised Cross Validation smoothing spline. Theia3D uses deep learning techniques to identify and track various anatomical features from the eight camera views. Inverse kinematics was also performed within Theia3D to obtain the pose of each body segment where the pelvis has 6 degrees of freedom and the hip, knee and ankle have 3 degrees of freedom. These data as well as the marker-based data were exported to Visual3D.

Joint angles of the pelvis, hip, knee and ankle in the sagittal, frontal and transverse planes as well as the foot progression angle were calculated. The kinetic parameters of interest were the internal moments of the hip, knee and ankle in the sagittal and frontal planes, all scaled to participant’s body mass. Sagittal powers of the hip, knee and ankle were also calculated for both models. All signals were time normalised to 101 data points of the gait cycle. Statistical analysis was carried out by means of Root Mean Square Error (RMSE) and Pearson’s correlation coefficient (CC). RMSE quantifies differences between courses^29^ where values above 5° can lead to clinically erroneous conclusion^30^. As per Heimsch et al.^31^, CC values were classified into very low (0-0.2), low (0.2–0.5), moderate (0.5–0.7) and high (0.7–0.9) where a positive high CC objectifies consistency between courses^32^. Combining both classifications, we have defined those joint angles with an RMSE > 5° and a very low correlation (< 0.2) to be non-comparable. Data normality was verified using the Shapiro-Wilk test.

A one-dimensional Statistical Parametric Mapping (1D-SPM) paired t-test was performed to determine differences between both systems (significance level α = 0.05). SPM was originally developed for statistical inference on continuous data such as neuroimaging data^33^. Biomechanics data such as kinetics and kinematics are also continuous data and are therefore suited for the use of 1D-SPM^34^. Inter-trial variability (ITV) was calculated for each parameter using the method described by Schwartz et al.^35^. In gait analysis, the degree of natural variability within a subject may taint any analysis designed to detect the effects of an intervention. The measurement and description of the inherent inter-trial variability is therefore of fundamental importance and must be known before drawing conclusions about the nature of a particular intervention, or in our case, the comparative analysis between two models. The statistical analysis was performed in Matlab™9.12.0.1956245 (R2022a) Update 2. 1D-SPM was carried out using the Matlab toolbox (https://spm1d.org).

## Results

The kinematics of six trials (1 left and 1 right gait cycle per trial) per subject were analysed. It was not possible to attain an equal number of kinetics since not all subjects, especially the younger ones, could hit the force plates cleanly or produce clean force strikes in all trials.

### Kinematics

**Fig. 2:**
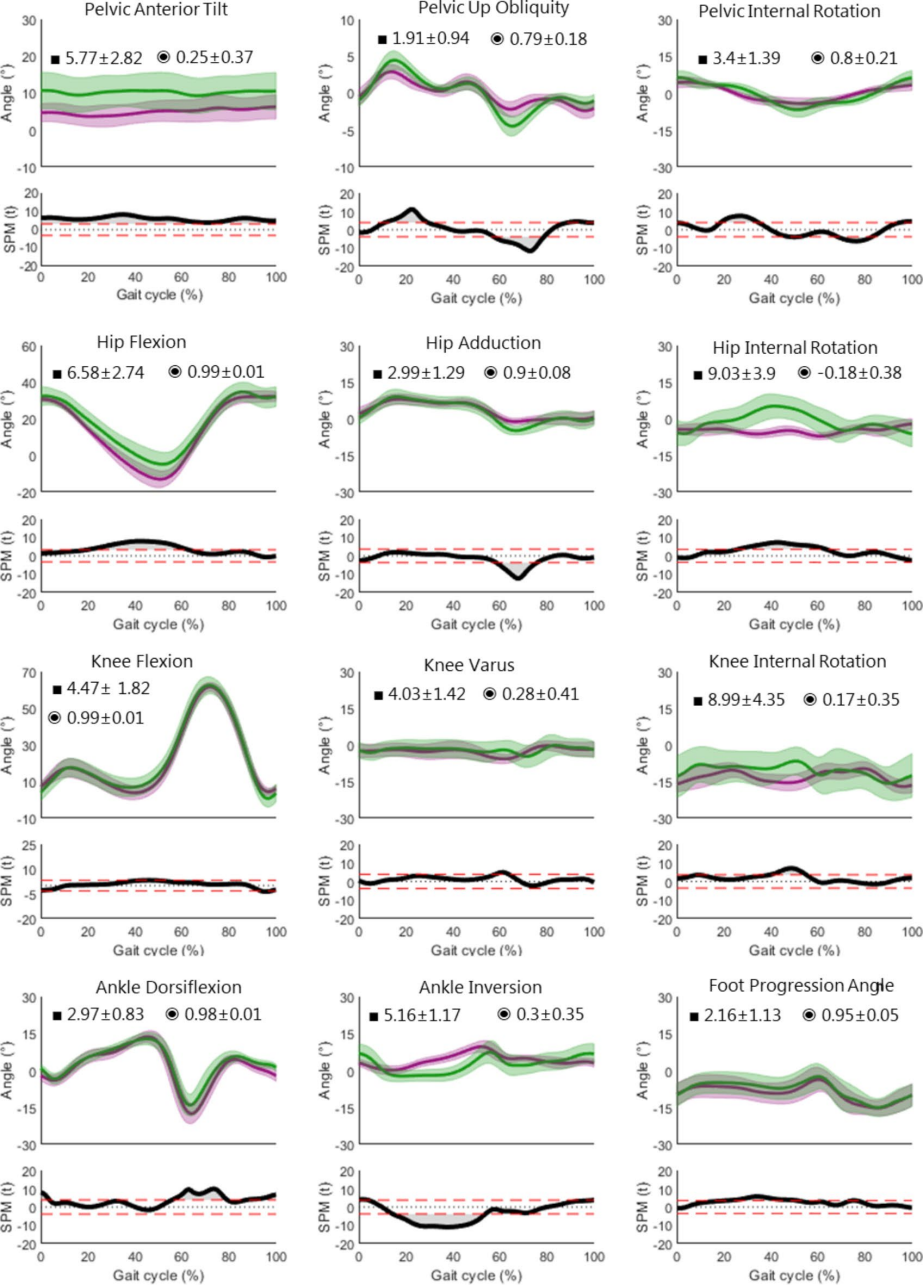
Physiological gait kinematics: Time normalised lower-body gait kinematics (± 1SD) and mean (bold) of 12 healthy subjects; marker-based CAST model (green) and Theia3D markerless model (purple). Average RMSE (■) and CC (◉) ± 1SD are displayed in each plot. 1D-SPM illustrating significant differences are plotted below each angle (α = 0.05).

Figure 2 shows the time-normalised mean curves with the corresponding 1^st^ standard deviation (± 1SD) bands of the markerless (purple) and marker-based (green) lower body kinematics superimposed on each other. The average RMSE (■) and CC (◉) with the ± 1SD are also displayed in each plot. 1D-SPM (α = 0.05) illustrates the significant differences between both systems.

Overall, the markerless data follows the same physiological trend as the marker-based data except in case of hip and knee internal rotation. There is a peak internal hip rotation at 50% of the gait cycle in the marker-based data whereas the markerless data shows a slight external rotation throughout the gait cycle. SD of the markerless hip and knee rotations are also smaller. The pelvic tilt has an offset during the entire gait cycle, hip flexion at 15–70%, knee flexion at 40–60%, ankle dorsiflexion at 50–100% and ankle inversion at 20–60%. Significant statistical differences are shown by 1D-SPM at 20% in pelvic obliquity, 30% pelvic tilt, 20–50% ankle inversion, 50% knee rotation, 40–60% hip flexion and rotation, 70% hip adduction and 60–80% ankle dorsiflexion. Most angles have a relatively low RMSE (< 5°) and high CC. The angles faring the poorest were the transverse plane hip and knee with an RMSE of approximately 9° and very low CC of approximately 0.2 and − 0.2 respectively. The knee varus, ankle inversion and pelvic tilt had a relatively good RMSE of 4–5° but a poor CC of 0.2–0.3. Another exception is the hip flexion with a poor RMSE of 6.6 and very good CC of 0.99. In summary, joint angles which are not comparable are internal hip and knee rotation.

Examining the ITV in Table 1, we see similarities between both models in terms of the amount of ITV. The ITV of the hip rotation, knee rotation and foot progression angle are markedly high (above 3°) in both models (marked in bold in Table 1). The other joint angles also have relatively similar but lower values between models (between 1° and 3°). The average ITV across all joints and all planes in both models is similar at about 2.5°.

**Fig. 3:**
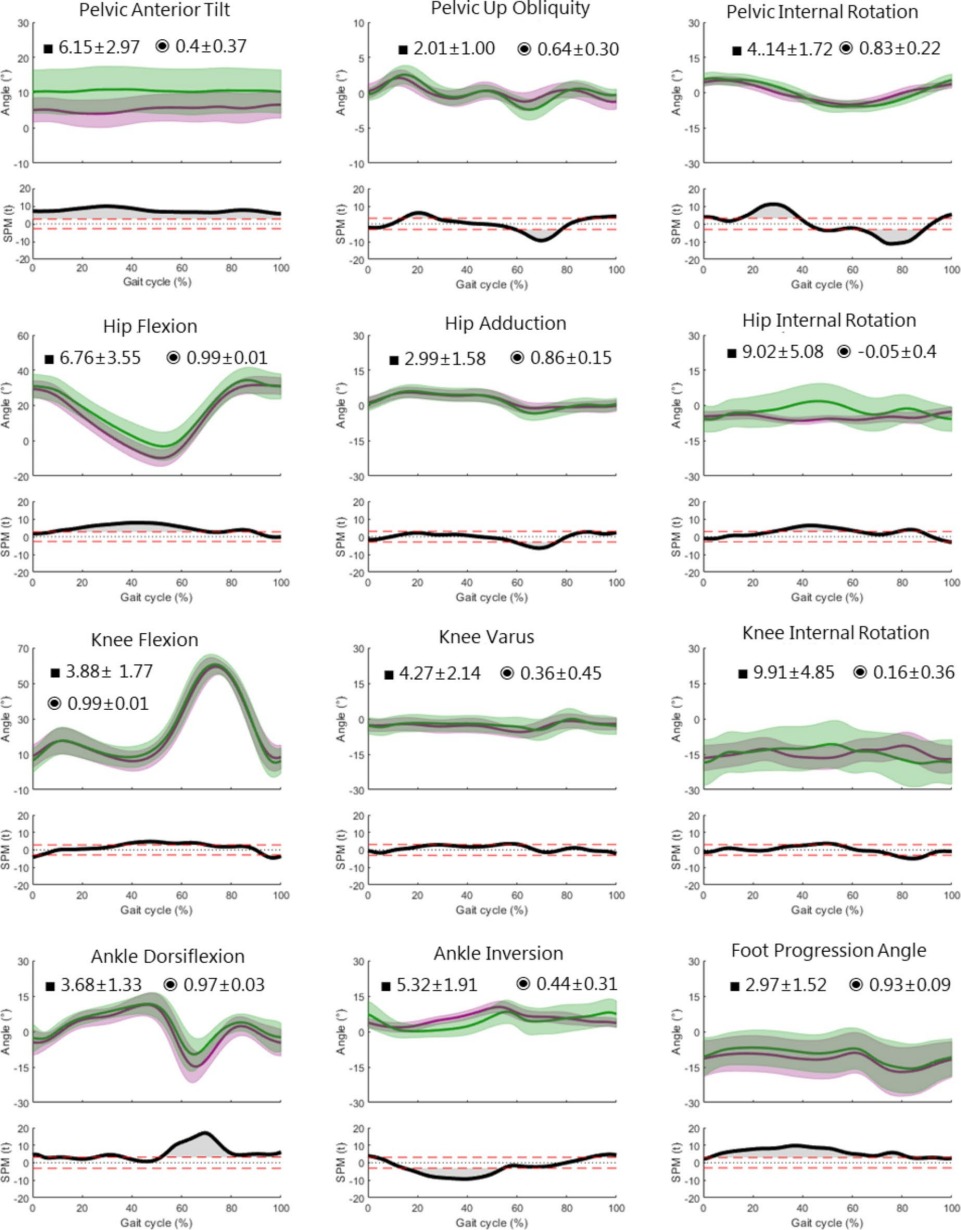
Pathological gait kinematics: Time normalised lower-body gait kinematics (± 1SD) and mean (bold) of 34 patients; marker-based CAST model (green) and Theia3D markerless model (purple). Average RMSE (■) and CC (◉) ± 1SD are displayed in each plot. 1D-SPM illustrating significant differences are plotted below each angle (α = 0.05).

Figure 3 shows the time normalised lower-body gait kinematics and statistical results of pathological gait. As expected in a group of patients with different walking disorders, one sees a larger dispersion corridor in both markerless and marker-based models especially as regards pelvic tilt, flexion and rotation of the hip and knee, ankle dorsiflexion as well as foot progression angle. However, the spread of markerless internal hip rotation appears to be relatively unchanged. This is objectively corroborated with increased RMSE and SD in all joint angles except for knee flexion and hip rotation. As with physiological gait, the joint angles with the highest RMSEs continue to be internal hip and knee rotation (~ 9°) followed by pelvic tilt and hip flexion (~ 6°). The internal hip and knee rotations also exhibit very low correlations, making these two joint angles non-comparable.

Looking at the SPM results, one sees that the statistically significant differences whilst appearing at similar time intervals are higher than in physiological kinematics. These higher statistical differences are especially true for pelvic rotation, ankle plantarflexion and foot progression.

Looking at the ITV results, we see a marked increase across all joints and planes in both models leading to an average of about 3.5°. The number of joint angles with higher ITV greater than 3° (marked in bold in Table 1) is seen not only in hip rotation, knee rotations and foot progression angle as with physiological kinematics but also in pelvic rotation, hip flexion, knee flexion and ankle dorsiflexion.

**Table 1:** Inter trial variability (ITV) of physiological and pathological lower-body gait kinematics and kinetics of the Theia3D markerless and CAST marker-based models.

Inter-trial variability (°)	Physiological gait		Pathological gait	
	Markerless	Marker-based	Markerless	Marker-based
Pelvic anterior tilt	1.95	1.11	2.12	1.65
Pelvic upward obliquity	1.33	1.69	1.58	1.96
Pelvic internal rotation	2.82	2.78	4.78	5.05
Hip flexion	2.85	2.02	**3.76**	2.99
Hip adduction	1.49	2.36	2.24	2.82
Hip internal rotation	**3.11**	**4.78**	**3.52**	**5.22**
Knee flexion	2.88	2.8	**4.17**	**4.07**
Knee varus	1.94	2.01	2.6	2.13
Knee internal rotation	**3.87**	**5.18**	**5.47**	**4.51**
Ankle dorsiflexion	2.05	1.81	3.36	3.19
Ankle inversion	1.77	2.12	2.41	3.2
Foot internal progression	**3.61**	**3.46**	**5.1**	**5.15**
Hip extensor moment	0.09	0.07	0.1	0.08
Hip valgus moment	0.08	0.06	0.1	0.08
Knee extensor moment	0.07	0.06	0.09	0.08
Knee valgus moment	0.06	0.04	0.07	0.05
Ankle plantarflexion moment	0.05	0.05	0.1	0.09
Hip sagittal power generation	0.17	0.12	0.17	0.14
Knee sagittal power generation	0.16	0.15	0.18	0.17
Ankle sagittal power generation	0.16	0.13	0.23	0.18

Values of ITV above 3° are marked in bold.

### Kinetics

**Fig. 4:**
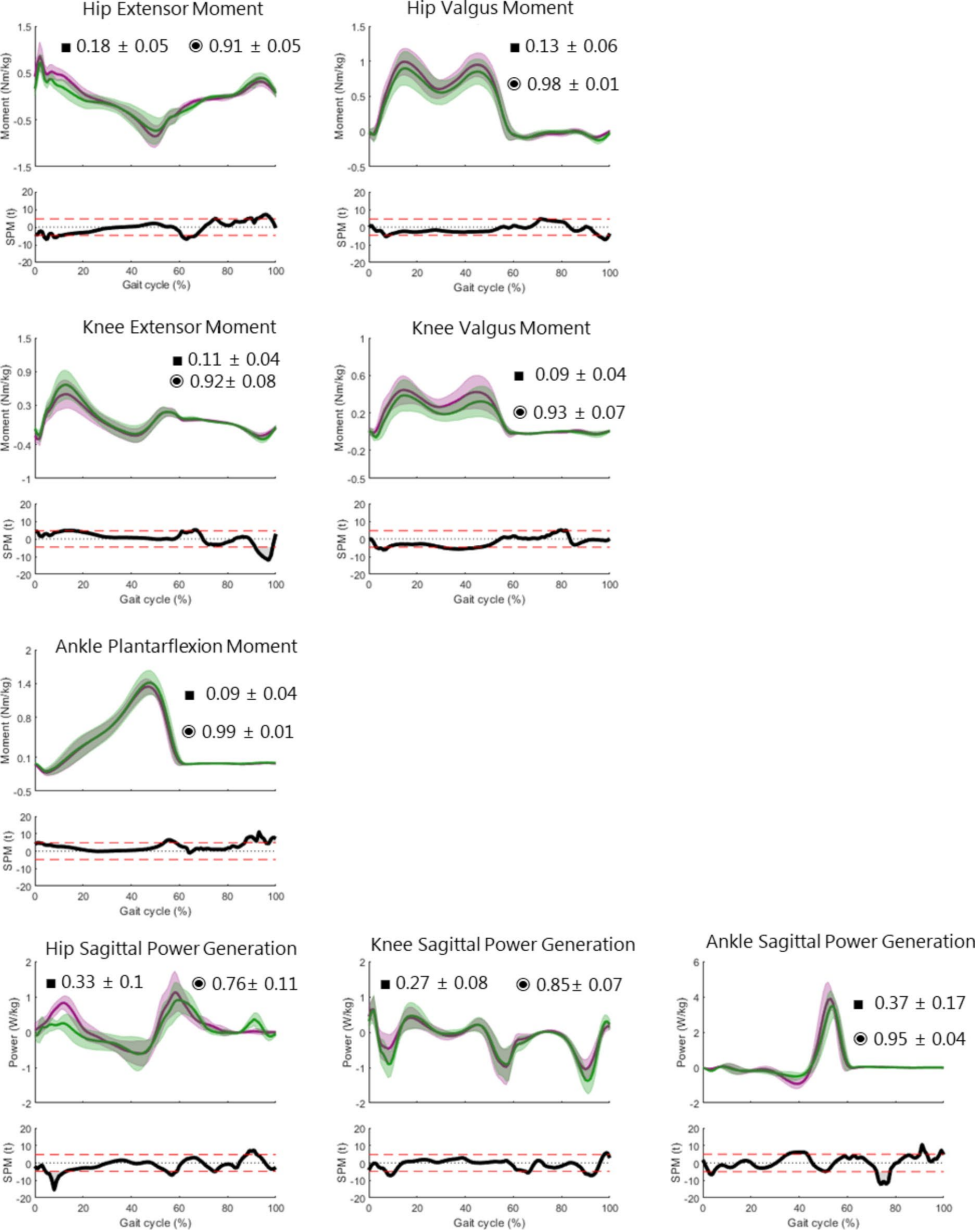
Physiological gait kinetics: Time normalised internal moments and powers (± 1SD) and mean (bold) of 12 healthy subjects; marker-based CAST model (green) and Theia3D markerless model (purple). Average RMSE (■) and CC (◉) ± 1SD are displayed in each plot. 1D-SPM illustrating significant differences (α = 0.05) are plotted below each angle.

The kinetic results for the physiological gait are shown in Fig. 4 with time-normalised mean curves and ± 1SD bands of markerless (purple) and marker-based (green) internal moments and powers superimposed on each other. The average RMSE (■) and CC (◉) with the ± 1SD are also displayed in each plot. 1D-SPM (α = 0.05) illustrates the significant differences between both systems. As with the kinematics, visual observation shows a good agreement between the markerless and marker-based kinetics. The numerical comparison of both models reveals a combination of good RMSE and CC values for all signals (RMSE between 0.1 and 0.4Nm/kg, CC above 0.8).

There are slight offsets especially between the peaks in the frontal plane. At 10% of the gait cycle, the hip extensor moment is slightly overestimated and knee extensor moment slightly underestimated compared to the normative data. These differences are reflected in the power curves. ITV values of kinetics in both models are extremely good, the highest value being 0.2° in almost all power curves for both models and the average of all kinetic moments and powers being 0.1° (Table 1).

**Fig. 5:**
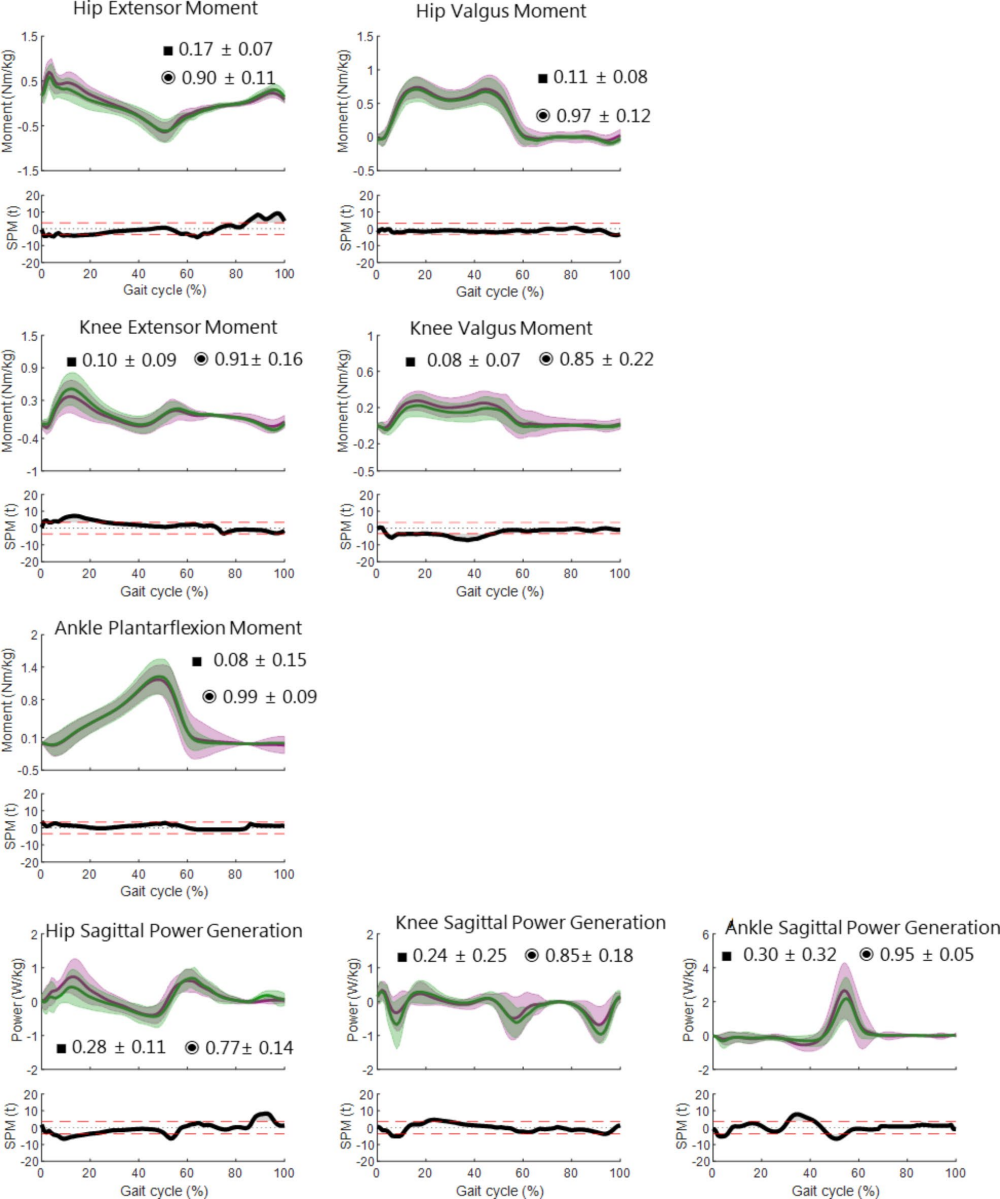
Pathological gait kinetics: Time normalised internal moments and powers (± 1SD) and mean (bold) of 34 patients; marker-based CAST model (green) and Theia3D markerless model (purple). Average RMSE (■) and CC (◉) ± 1SD are displayed in each plot. 1D-SPM illustrating significant differences (α = 0.05) are plotted below each angle.

Similar to pathological kinematics, the initial impression that one gets from visual observation of the pathological kinetics is a higher spread of the data in both models but otherwise there seems to be a good trend between both models (Fig. 5). The double-bump patterns of the hip and knee valgus moments in general are also reduced. The ITV values for both models are slightly increased but maintain a similar consistency as with physiological ITV with the maximum continuing to be circa 0.2° for markerless and marker-based models (Table 1).

### Interpretation of a clinical case study

**Fig. 6:**
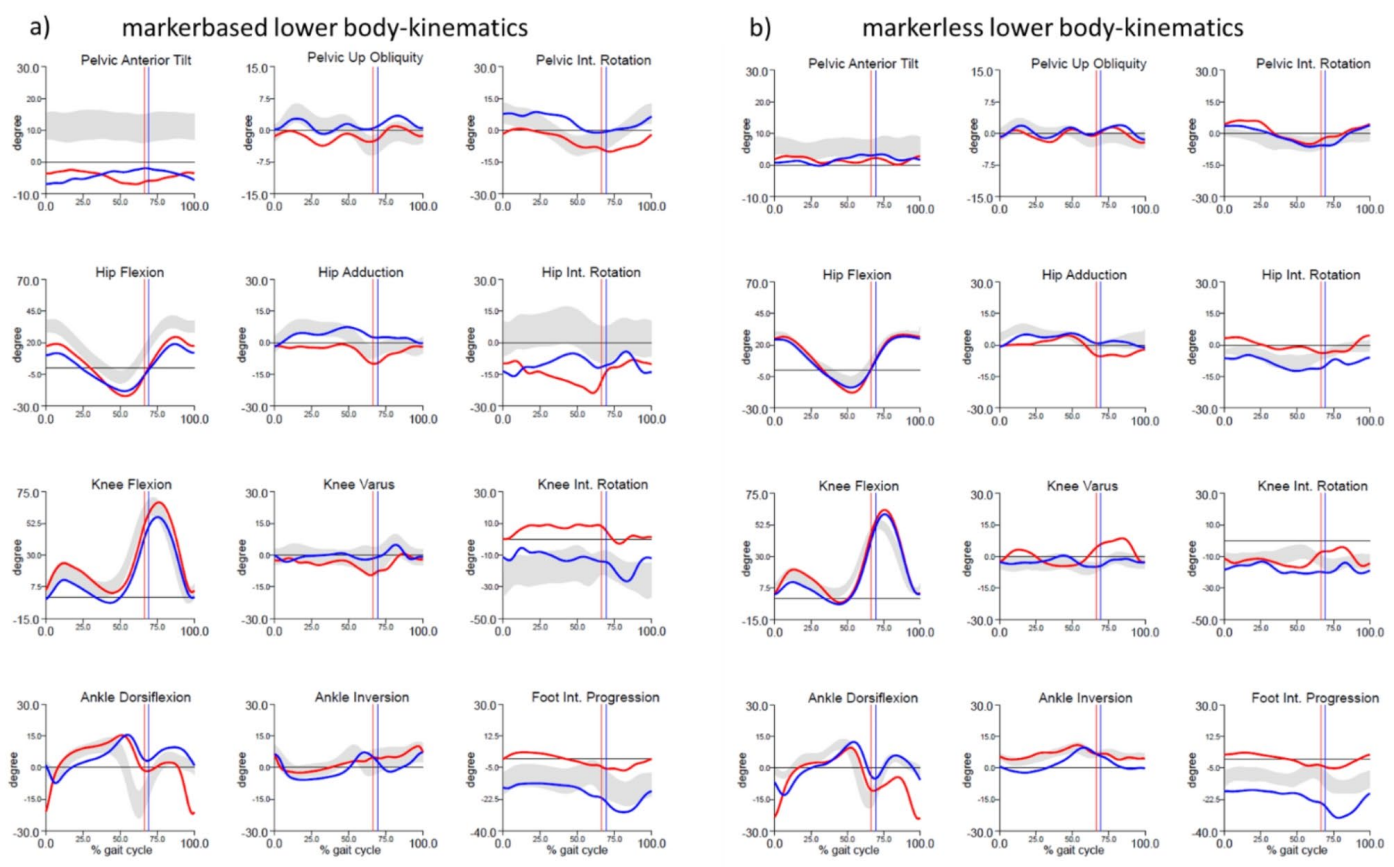
Averaged lower-body gait kinematics (red = left body side and blue = right body side) of a 15 year old patient diagnosed with Hereditary Motor Sensory Neuropathy (HSMN) where (**a**) depicts the marker-based model and (**b**) the markerless model. The vertical lines indicate the end of the stance phase and start of the swing phase (toe-off).

Normative data (± 1SD) of the healthy group were established separately for marker-based and markerless models. The kinematics of each patient was plotted against the normative data for both models (exemplarily shown in Fig. 6). The reason behind this is not to focus on the absolute differences between markerless and marker-based data per se, but to evaluate whether one arrives at the same clinical interpretation for the same patient whilst using different systems. The knee flexion/extension and ankle dorsiflexion/plantarflexion graphs displayed similar trends as far as description of the patient data went. However, almost all patients, with the exception of three displayed different outcomes in hip and/or knee rotation.

To highlight the stark differences in in the transverse plane, we present a patient with Hereditary Motor Sensory Neuropathy (HSMN) (Fig. 6) walking with a severe drop-foot on the left side. Once again, it is important to note that the patient graphs are superimposed with the normative data of the corresponding model.

In the sagittal plane, the markerless pelvic tilt is slightly posterior but not as marked as its marker-based counterpart. This difference is also visible in the hip flexion graphs. Both knee flexion graphs show a similar outcome in terms of slight hyperextension at about 50% gait cycle especially on the right side. The severe left drop foot (red) can also be seen in both models with large plantarflexion from 75% to about 10% gait cycle.

The frontal plane has small differences but serves us with similar interpretation of deviations from the normative bands.

The transverse plane reveals similar interpretation of foot progression (negative on the left foot, and positive on the right foot). The slight pelvic external rotation toward the left side in the marker-based model is rather a normalised markerless pelvic rotation. The biggest discrepancy however, is seen in the left hip and knee rotation graphs. The marker-based model measures a large external hip rotation which is compensatory for the drop foot in order to achieve foot clearance in the swing phase. This compensation mechanism is also evident in the video analysis of the patient. The markerless model however measures a continuous slight internal rotation which is not indicative of the actual discrepancy. In summary, the differences in the hip rotation between both systems leads to a different clinical interpretation depending on which system is used for analysis.

## Discussion

The present study aimed at evaluating the validity of lower body gait kinematics and kinetics as measured by the Theia3D markerless system compared to the marker-based CAST model in a group of adults and children demonstrating healthy and pathological walking patterns.

A high positive correlation coefficient, which objectifies the consistency between the courses^32,36^ was defined as a necessary criterion for good comparability, as the course is decisive in clinical interpretation^37,38^. The RMSE quantifies differences in the courses^29^. According to McGinley et al.^30^errors between 2°and 5°are considered relevant for clinical interpretation, whereby they can lead to clinically erroneous conclusions, especially above 5°. Therefore, if the necessary criterion is met, RMSE below 5° are categorised as being readily comparable irrespective of significant differences. If the RMSE is higher than 5 (and there are also many significant differences (> 25%) between the data), the data in this study are not considered comparable.

### Kinematics

Overall, the markerless system demonstrated similar gait patterns except in case of the internal hip and knee rotations, which were concluded to be non-comparable. Significant underestimation of the pelvic anterior tilt was seen. Earlier studies on Theia3D have not focused on the pelvic segment. Theia3D not being open-source, it is difficult to elaborate on the differences found with marker-based systems and highlight that future studies should focus on the pelvis definition. The downward shift in the pelvic tilt was propagated into the hip joint resulting in a reduced hip flexion. Ankle dorsiflexion and inversion were significantly different during the swing phase and stance phase, respectively. It must be emphasized that marker-based motion capture is by no means a validated benchmark. The true accuracy of any system can only be established by comparing to a gold standard such as bi-planar videography.

Comparing the results of this study with those of Kanko et al.^22^ one can see a similar spread of the data, markerless being less than marker-based, especially in the hip and knee rotational angles. Kanko et al. reported an offset in hip flexion during almost all phases of the gait cycle whereas our data presents an offset from mid to late stance. Increased markerless ankle inversion from 20 to 60% of the gait cycle is seen in both studies. The relatively constant slight externally rotation (5°) of markerless hip as well as the difference in the trends between markerless and marker-based rotation especially at 40% of the gait cycle are seen in both studies. This difference is more conspicuous in our study which could be related to the use of different marker-based models (CAST versus Vicon PiG) and/or the comparatively low sample size in our study (12 versus 30 subjects). Pelvic angles were not presented in their study and a comparison therefore not possible.

One should also keep in mind that both studies used different versions (v2021.1.0.1450 and v2022.1.0.2309) of Theia3D for data processing. Pose estimation in Theia3D has been continuously improved in terms of number of features, number of images and quality of images, contributing to improved localization of anatomical points. There have also been improvements in quality assurance, segment definitions and inverse kinematics in the new Theia3D versions.

McGuirk et al.^24^ presented the lower-body markerless kinematics of 28 healthy individuals (adults and children) albeit without pelvic tracking. Here we find similarities to this study in terms of the 20° maximum hip extension and similar values of peak hip abduction in initial swing. The hip rotation in both studies also follows a similar trend. External knee rotation is higher at the beginning of the stance phase in comparison to the data of this study, then levelling off at similar values during the rest of the gait cycle. Again, one should bear in mind that the current study uses a more recent version of Theia3D (v2021.20.1675) compared to McGuirk et al. and therefore uses more anatomical landmarks for pose estimation of the skeleton.

When considering the kinematics of subjects with gait pathologies, the differences and similarities between the marker-based and markerless graphs were similar to those in healthy subjects. Wren et al.^28^ (Theia3D v2021.2.0.1675) also reported similar outcomes in a groups of thirty-six patients with a range of orthopaedic and neuro-orthopaedic diagnoses where an unknown number of left strides from one of more walking trials per subject were analysed. In our study, 6 gait cycles per side, per subject were analysed due to the constraints of time and patient ability/compliance in a clinical setting. The overall low variability in the dispersion corridor of the pathological group in our study suggests a certain homogeneity which may be attributed to the mixture of orthopaedic (36%) with neuro-orthopaedic disorders (64%).

#### Clinical case study

However, when considering if the clinical interpretation of the patient data is similar across both models, this study concludes that the hip and knee rotation graphs produce different outcomes in almost all patients. In the clinical case study presented (Fig. 6), the severely increased pelvic posterior tilt and hip extension as shown by the marker-based system is presented within the normal range of the markerless system. The clinical interpretation is starkly different between both systems due to extreme differences in the hip rotation, thereby leading to different outcomes.

### Kinetics

The results of the kinetics are partly consistent with those of Tang et al.^25^ who examined 16 subjects using the same version of Theia3D for post-processing as this study (v2022.1.0.2309). We also found greater peak magnitudes of knee valgus moment and in the power graphs, but not in the ankle plantarflexion moment. The changes could also be attributed to the different movement analysed i.e. treadmill running versus overground walking and therefore should be handled with care.

That marker-based motion capture is not without its failings is a known fact. Intra- and inter-operator variability in marker placement are major contributors to overall reproducibility in gait measurements^39,40^. This could partly explain the higher spread of some of the marker-based curves. Soft tissue artefact is a source of error that depends on the segment under analysis^41^. When propagated, it creates critical errors in the frontal and transverse planes of movement^10^. It should also be noted that significant differences in gait kinematics are evident between different marker-based models especially in the axial rotations of the thigh^42^ thereby proving the lack of consensus especially in this area.

### ITV

The ITV in healthy subjects showed higher scores (3°-5°) for hip and knee rotation and foot progression angles. Both rotational ITVs were higher in case of the marker-based data compared to the markerless data. Pathological ITV incremented for all three joint angles. In addition, higher ITVs were also seen for pelvic internal rotation, hip flexion and knee flexion. Wren et al. reported sometimes having greater ITV for the markerless compared with the marker-based system but lack of synchronisation of both systems and a lack of objective summarisation of the findings makes a direct comparison with our study difficult^28^.

### Theia3D markerless vs. instrumented marker-based system

On the one hand, Theia3D overcomes the drawbacks of marker-based motion capture in terms of minimal clothing, preparation time and marker loss or occlusion. The problem of skin artefacts is yet to be investigated. The volume of data generated is however significantly larger than that in marker-based systems, making it potentially expensive to archive. Processing time is also reasonably higher which does not allow for quality assurance during data capture^28^. This is especially crucial in a clinical setting where a fast-track report is created to ensure adequate data quality before a patient leaves the lab. Other issues like accuracy, sensitivity to environmental conditions (light, number of people in the camera field etc.), effect of different clothing^43^ are also currently being investigated.

Further developments of markerless systems, i.e. 3D multi-camera systems, could include but are not limited to: improvements to the neural network architecture predicting the anatomical points, improving the definition of the human model based on these predictions, collecting more training data, improving the labelling accuracy of the training data and finally using different neural network approaches such as predicting the segment pose instead of anatomical points^44^.

### Theia3D markerless vs. other markerless systems

Direct comparisons with other studies must be exercised with caution because of dissimilarities between biomechanical models^42^, different age groups, different movements assessed and limited joint planar angles assessed. Some studies have examined only the knee flexion angle and found this measure to have similar results between both systems^45,46^. Harsted et al. examined various functional movements in pre-schoolers by means of “The Captury” markerless system and a Vicon marker-based system^47^. They found invalid hip flexion measures, substantial knee flexion differences contradictory to this study whereas rotational measures were not assessed^47^. The results of our study align with Sandau et al. who found similar measures in sagittal plane and hip frontal plane gait kinematics of 10 healthy subjects but less reliability in rotations and ankle inversion^48^. However, the pelvis segment was not assessed by Sandau et al.^48^. Based on simultaneous data collection in gait, Ceseracciu et al. also reported that hip joint centre locations were the most incorrect whereas sagittal knee kinematic estimation was better^49^. These results are in concurrence with those of our study.

## Conclusion

The findings of the full assessment of the markerless technology from this study suggests that the measurements by the Theia3D markerless system are not interchangeable with the marker-based measures. But the results infer that Theia3D offers great potential in gait analysis. It shows similar deviations between markerless and marker-based systems in both healthy and clinical patients except in hip and knee rotations which are non-comparable. In summary, the study highlights areas where clinical practitioners and researchers can be confident, as well as points out where they should be cautious when interpreting standard gait analysis results including kinematics and kinetics. We suggest extensive testing be conducted before this system can be used for clinical decision-making, particularly expanding the evaluation to the upper body and also comparing with other marker-based models. Although markerless systems are destined to replace marker-based systems in certain applications, the latter will still be necessary for certain other applications, such as multi-segment foot and spine models.

In addition to gait analysis, other research fields are opening up for the use of markerless systems, e.g. movement analysis in sports^50,51^, during human machine interactions^52^, in musculoskeletal biomechanics^53^ or even in animals^54,55^ where the setting of markers is difficult or impossible.

## Electronic supplementary material

Below is the link to the electronic supplementary material.

Supplementary Material 1

## Data Availability

The datasets generated during and/or analyzed during the current study are available from the corresponding author on reasonable request.

## References

[1] Gage, J. R. *Gait Analysis in Cerebral Palsy* (Mac Keith, 2004).

[2] Robertson, D. G. E., Caldwell, G. E., Hamill, J., Kamen, G. & Whittlesey, S. N. *Research Methods in Biomechanics* 2nd edn (Human Kinetics, 2014).

[3] Winter, D. A. *Biomechanics and Motor Control of Human Gait* 2nd edn (John Wiley & Sons, Inc., 1990).

[4] Stagni, R., Leardini, A., Cappozzo, A., Benedetti, M. G. & Cappello, A. Effects of hip joint centre mislocation on gait analysis results. *J. Biomech.* 1479–1487. (2000). 10.1016/s0021-9290(00)00093-2 10940407

[5] Ewa Szczerbik, M. K. The influence of knee marker placement error on evaluation of gait kinematic parameters. *Acta Bioeng. Biomech.* **13** (2011). 22098124

[6] Groen, B. E., Geurts, M., Nienhuis, B. & Duysens, J. Sensitivity of the OLGA and VCM models to erroneous marker placement: effects on 3D-gait kinematics. *Gait Posture*. **35**, 517–521. 10.1016/j.gaitpost.2011.11.019 (2012). 22245226 10.1016/j.gaitpost.2011.11.019

[7] Nazareth, A., Mueske, N. M. & Wren, T. A. L. Effect of Tibia marker Placement on kinematics in Pathological Gait. *J. Appl. Biomech.* **32**, 603–607. 10.1123/jab.2015-0219 (2016). 27619915 10.1123/jab.2015-0219 PMC6373764

[8] Kainz, H. et al. Effects of hip joint centre mislocation on gait kinematics of children with cerebral palsy calculated using patient-specific direct and inverse kinematic models. *Gait Posture*. **57**, 154–160. 10.1016/j.gaitpost.2017.06.002 (2017). 28641160 10.1016/j.gaitpost.2017.06.002

[9] Cappozzo, A., Catani, F., Della Croce, U. & Leardini, A. Position and orientation in space of bones during movement: anatomical frame definition and determination. *Clin. Biomech. Elsevier Ltd* 171–178. (1995). 10.1016/0268-0033(95)91394-t 11415549

[10] Stagni, R., Fantozzi, S., Cappello, A. & Leardini, A. Quantification of soft tissue artefact in motion analysis by combining 3D fluoroscopy and stereophotogrammetry: a study on two subjects. *Clin. Biomech. (Bristol, Avon)*. **20**, 320–329. 10.1016/j.clinbiomech.2004.11.012 (2005). 15698706 10.1016/j.clinbiomech.2004.11.012

[11] Rouhandeh, A., Joslin, C., Qu, Z. & Ono, Y. Quantification of soft tissue artefacts using motion capture data and Ultrasound depth measurements. *Int. J. Bioeng. Life Sci.* **8**, 334–338 (2014). 10.1109/EMBC.2014.6944585 25570953

[12] Leardini, A. et al. Kinematic models of lower limb joints for musculo-skeletal modelling and optimization in gait analysis. *J. Biomech.* **62**, 77–86. 10.1016/j.jbiomech.2017.04.029 (2017). 28601242 10.1016/j.jbiomech.2017.04.029

[13] Mündermann, L., Corazza, S. & Andriacchi, T. P. The evolution of methods for the capture of human movement leading to markerless motion capture for biomechanical applications. *J. Neuroeng. Rehab.* **3**, 6. 10.1186/1743-0003-3-6 (2006). 10.1186/1743-0003-3-6 PMC1513229 16539701

[14] Cronin, J. N. Using deep neural networks for kinematic analysis: challenges and opportunities. *J. Biomech.* **123**, 110460. 10.1016/j.jbiomech.2021.110460 (2021). 34029787 10.1016/j.jbiomech.2021.110460

[15] Drazan, J. F., Phillips, W. T., Seethapathi, N., Hullfish, T. J. & Baxter, J. R. Moving outside the lab: markerless motion capture accurately quantifies sagittal plane kinematics during the vertical jump. 10.1016/j.jbiomech.2021.110547 (2021). 10.1016/j.jbiomech.2021.110547 PMC8640714 34175570

[16] Sato, K., Nagashima, Y., Mano, T., Iwata, A. & Toda, T. Quantifying normal and parkinsonian gait features from home movies: practical application of a deep learning-based 2D pose estimator. *PloS One*. **14**, e0223549. 10.1371/journal.pone.0223549 (2019). 31725754 10.1371/journal.pone.0223549 PMC6855634

[17] Wade, L., Needham, L., McGuigan, P. & Bilzon, J. Applications and limitations of current markerless motion capture methods for clinical gait biomechanics. *PeerJ* **10**, e12995. 10.7717/peerj.12995 (2022). 35237469 10.7717/peerj.12995 PMC8884063

[18] Mathis, A., Schneider, S., Lauer, J. & Mathis, M. W. A primer on Motion capture with deep learning: principles, pitfalls, and perspectives. *Neuron* **108**, 44–65. 10.1016/j.neuron.2020.09.017 (2020). 33058765 10.1016/j.neuron.2020.09.017

[19] Nakano, N. et al. Evaluation of 3D Markerless Motion capture Accuracy using OpenPose with multiple video cameras. *Front. Sports Act. Living*. **2** (50). 10.3389/fspor.2020.00050 (2020). 10.3389/fspor.2020.00050 PMC7739760 33345042

[20] Zago, M. et al. 3D Tracking of Human Motion using Visual Skeletonization and Stereoscopic Vision. *Front. Bioeng. Biotechnol.* **8**, 181. 10.3389/fbioe.2020.00181 (2020). 32195243 10.3389/fbioe.2020.00181 PMC7066370

[21] Kanko, R. M., Laende, E., Selbie, W. S. & Deluzio, K. J. Inter-session repeatability of markerless motion capture gait kinematics. *J. Biomech.* **121**, 110422. 10.1016/j.jbiomech.2021.110422 (2021). 33873117 10.1016/j.jbiomech.2021.110422

[22] Kanko, R. M., Laende, E. K., Davis, E. M., Selbie, W. S. & Deluzio, K. J. Concurrent assessment of gait kinematics using marker-based and markerless motion capture. *J. Biomech.* **127**, 110665. 10.1016/j.jbiomech.2021.110665 (2021). 34380101 10.1016/j.jbiomech.2021.110665

[23] Kanko, R. M. et al. Assessment of spatiotemporal gait parameters using a deep learning algorithm-based markerless motion capture system. *J. Biomech.* **122**, 110414. 10.1016/j.jbiomech.2021.110414 (2021). 10.1016/j.jbiomech.2021.110414 33915475

[24] McGuirk, T. E., Perry, E. S., Sihanath, W. B., Riazati, S. & Patten, C. Feasibility of Markerless Motion capture for three-dimensional Gait Assessment in Community settings. *Front. Hum. Neurosci.* **16**, 867485. 10.3389/fnhum.2022.867485 (2022). 35754772 10.3389/fnhum.2022.867485 PMC9224754

[25] Tang, H., Pan, J., Munkasy, B., Duffy, K. & Li, L. Comparison of Lower Extremity Joint Moment and Power Estimated by Markerless and Marker-Based Systems during Treadmill Running. *Bioengineering (Basel, Switzerland)* **9**. 10.3390/bioengineering9100574 (2022). 10.3390/bioengineering9100574 PMC9598493 36290542

[26] Ito, N. et al. Markerless motion capture: what clinician-scientists need to know right now. *JSAMS plus*. **1**10.1016/j.jsampl.2022.100001 (2022). 10.1016/j.jsampl.2022.100001 PMC9699317 36438718

[27] Lam, W. W. T., Tang, Y. M. & Fong, K. N. K. A systematic review of the applications of markerless motion capture (MMC) technology for clinical measurement in rehabilitation. *J. Neuroeng. Rehabil.* **20**10.1186/s12984-023-01186-9 (2023). 10.1186/s12984-023-01186-9 PMC10155325 37131238

[28] Wren, T. A. L., Isakov, P. & Rethlefsen, S. A. Comparison of kinematics between Theia markerless and conventional marker-based gait analysis in clinical patients. *Gait Posture*. **104**, 9–14. 10.1016/j.gaitpost.2023.05.029 (2023). 37285635 10.1016/j.gaitpost.2023.05.029

[29] Flux, E. et al. The human body model versus conventional gait models for kinematic gait analysis in children with cerebral palsy. *Hum. Mov. Sci.* **70**, 102585. 10.1016/j.humov.2020.102585 (2020). 32217202 10.1016/j.humov.2020.102585

[30] McGinley, J. L., Baker, R., Wolfe, R. & Morris, M. E. The reliability of three-dimensional kinematic gait measurements: a systematic review. *Gait Posture*. **29**, 360–369. 10.1016/j.gaitpost.2008.09.003 (2009). 19013070 10.1016/j.gaitpost.2008.09.003

[31] Heimsch, F., Niederer, R. & Zöfel, P. *Statistik Im Klartext. Für Psychologen, Wirtschafts- und Sozialwissenschaftler* 2nd edn (Pearson Studium, 2018).

[32] Benedetti, M. G., Merlo, A. & Leardini, A. Inter-laboratory consistency of gait analysis measurements. *Gait Posture*. **38**, 934–939. 10.1016/j.gaitpost.2013.04.022 (2013). 23711987 10.1016/j.gaitpost.2013.04.022

[33] Friston, K. J., Ashburner, J. T., Kiebel, S. J., Nichols, T. E. & Perry, W. D. *Statistical Parametric Mapping. The Analysis of Funtional Brain Images* 1st edn (Elsevier/Academic, 2007).

[34] Pataky, C. T. Generalized n-dimensional biomechanical field analysis using statistical parametric mapping. *J. Biomech.* **43**, 1976–1982. 10.1016/j.jbiomech.2010.03.008 (2010). 20434726 10.1016/j.jbiomech.2010.03.008

[35] Schwartz, M. H., Trost, J. P. & Wervey, R. A. Measurement and management of errors in quantitative gait data. *Gait Posture*. **20**, 196–203. 10.1016/j.gaitpost.2003.09.011 (2004). 15336291 10.1016/j.gaitpost.2003.09.011

[36] Ferrari, A. et al. Quantitative comparison of five current protocols in gait analysis. *Gait Posture*. **28**, 207–216. 10.1016/j.gaitpost.2007.11.009 (2008). 18206374 10.1016/j.gaitpost.2007.11.009

[37] Müller, B. et al. (eds) *Handbook of Human Motion* (Springer International Publishing, 2016).

[38] Sangeux, M., Passmore, E., Graham, H. K. & Tirosh, O. The gait standard deviation, a single measure of kinematic variability. *Gait Posture*. **46**, 194–200. 10.1016/j.gaitpost.2016.03.015 (2016). 27131201 10.1016/j.gaitpost.2016.03.015

[39] Scalona, E. et al. Inter-laboratory and inter-operator reproducibility in gait analysis measurements in pediatric subjects. *Int. Biomech.* **6**, 19–33. 10.1080/23335432.2019.1621205 (2019). 34042002 10.1080/23335432.2019.1621205 PMC7857309

[40] Gorton, G. E., Hebert, D. A. & Gannotti, M. E. Assessment of the kinematic variability among 12 motion analysis laboratories. *Gait Posture*. **29**, 398–402. 10.1016/j.gaitpost.2008.10.060 (2009). 19056271 10.1016/j.gaitpost.2008.10.060

[41] Peters, A., Galna, B., Sangeux, M., Morris, M. & Baker, R. Quantification of soft tissue artifact in lower limb human motion analysis: a systematic review. *Gait Posture*. **31**, 1–8. 10.1016/j.gaitpost.2009.09.004 (2010). 19853455 10.1016/j.gaitpost.2009.09.004

[42] Collins, T. D., Ghoussayni, S. N., Ewins, D. J. & Kent, J. A. A six degrees-of-freedom marker set for gait analysis: repeatability and comparison with a modified Helen Hayes set. *Gait Posture*. **30**, 173–180. 10.1016/j.gaitpost.2009.04.004 (2009). 19473844 10.1016/j.gaitpost.2009.04.004

[43] Kanko, R. M., Outerleys, J. B., Laende, E. K., Selbie, W. S. & Deluzio, K. J. Comparison of concurrent and asynchronous running kinematics and kinetics from marker-based and Markerless Motion capture under varying Clothing conditions. *J. Appl. Biomech.* **40**, 129–137. 10.1123/jab.2023-0069 (2024). 38237574 10.1123/jab.2023-0069

[44] Bittner, M. et al. Towards Single Camera Human 3D-Kinematics. *Sensors (Basel, Switzerland)* **23**. 10.3390/s23010341 (2022). 10.3390/s23010341 PMC9823525 36616937

[45] Perrott, M. A., Pizzari, T., Cook, J. & McClelland, J. A. Comparison of lower limb and trunk kinematics between markerless and marker-based motion capture systems. *Gait Posture*. **52**, 57–61. 10.1016/j.gaitpost.2016.10.020 (2017). 27871019 10.1016/j.gaitpost.2016.10.020

[46] Pantzar-Castilla, E. et al. Knee joint sagittal plane movement in cerebral palsy: a comparative study of 2-dimensional markerless video and 3-dimensional gait analysis. *Acta Orthop.* **89**, 656–661. 10.1080/17453674.2018.1525195 (2018). 30558517 10.1080/17453674.2018.1525195 PMC6300740

[47] Harsted, S., Holsgaard-Larsen, A., Hestbæk, L., Boyle, E. & Lauridsen, H. H. Concurrent validity of lower extremity kinematics and jump characteristics captured in pre-school children by a markerless 3D motion capture system. *Chiropr. Man. Ther.* **27**, 39. 10.1186/s12998-019-0261-z (2019). 10.1186/s12998-019-0261-z PMC6689331 31417672

[48] Sandau, M. et al. Markerless motion capture can provide reliable 3D gait kinematics in the sagittal and frontal plane. *Med. Eng. Phys.* **36**, 1168–1175. 10.1016/j.medengphy.2014.07.007 (2014). 25085672 10.1016/j.medengphy.2014.07.007

[49] Ceseracciu, E., Sawacha, Z. & Cobelli, C. Comparison of markerless and marker-based motion capture technologies through simultaneous data collection during gait: proof of concept. *PloS One*. **9**, e87640. 10.1371/journal.pone.0087640 (2014). 24595273 10.1371/journal.pone.0087640 PMC3942307

[50] Suo, X., Tang, W. & Li, Z. Motion Capture Technology in Sports Scenarios: A Survey. *Sensors (Basel, Switzerland)* 24. 10.3390/s24092947 (2024). 10.3390/s24092947 PMC11086331 38733052

[51] Held, S., Siebert, T. & Donath, L. 10% higher Rowing Power outputs after Flexion-Extension-Cycle compared to an isolated concentric contraction in Sub-elite rowers. *Front. Physiol.* **11**, 521. 10.3389/fphys.2020.00521 (2020). 32625103 10.3389/fphys.2020.00521 PMC7311752

[52] Kempter, F., Lantella, L., Stutzig, N., Fehr, J. & Siebert, T. Role of rotated Head postures on Volunteer Kinematics and muscle activity in Braking scenarios performed on a driving Simulator. *Ann. Biomed. Eng.* **51**, 771–782. 10.1007/s10439-022-03087-9 (2023). 36224484 10.1007/s10439-022-03087-9 PMC10023650

[53] Holzer, D., Paternoster, F. K., Hahn, D., Siebert, T. & Seiberl, W. Considerations on the human Achilles tendon moment arm for in vivo triceps surae muscle-tendon unit force estimates. *Sci. Rep.* **10**, 19559. 10.1038/s41598-020-76625-x (2020). 33177655 10.1038/s41598-020-76625-x PMC7658232

[54] Weihmann, T., Reinhardt, L., Weißing, K., Siebert, T. & Wipfler, B. Fast and powerful: Biomechanics and Bite forces of the mandibles in the American Cockroach Periplaneta americana. *PloS One*. **10**, e0141226. 10.1371/journal.pone.0141226 (2015). 26559671 10.1371/journal.pone.0141226 PMC4641686

[55] Kaya, M., Leonard, T. & Herzog, W. Coordination of medial gastrocnemius and soleus forces during cat locomotion. *J. Exp. Biol.* **206**, 3645–3655. 10.1242/jeb.00544 (2003). 12966056 10.1242/jeb.00544

