# Supplementary material for: A comparison of lower body gait kinematics and kinetics between Theia3D markerless and marker-based models in healthy subjects and clinical patients
Source: Sci Rep. 2024 Nov 25;14:29154. doi: 10.1038/s41598-024-80499-8 (PMC11589150; doi:10.1038/s41598-024-80499-8)
Supplement: Supplementary file 1 — Supplementary Material 1 [file 41598_2024_80499_MOESM1_ESM.docx]

| **Diagnosis** | **Nr. of patients** |
| --- | --- |
| Tetraparesis | 2 |
| Bilateral cerebral palsy | 2 |
| Unilateral cerebral palsy | 5 |
| Pes planovalgus | 10 |
| Pes cavovarus | 1 |
| Leg length difference | 1 |
| Pain | 1 |
| Tibialis posterior syndrome | 1 |
| Encephalopathy | 1 |
| Microcephaly | 1 |
| Leukemia | 1 |
| Hydrocephalus | 1 |
| Muscular dystrophy | 1 |
| Chondrosarcoma | 1 |
| Developmental delay | 2 |
| Hereditary sensory motor neuropathy | 2 |
| Osteochondrosis | 1 |

Supplementary Table S1: Distribution of diagnoses of 34 patients with orthopaedic and/or neuro-orthopaedic disorders
